# Quercetin Liposomal Nanoformulation for Ischemia and Reperfusion Injury Treatment

**DOI:** 10.3390/pharmaceutics14010104

**Published:** 2022-01-03

**Authors:** Margarida Ferreira-Silva, Catarina Faria-Silva, Manuela C. Carvalheiro, Sandra Simões, H. Susana Marinho, Paulo Marcelino, Maria Celeste Campos, Josbert M. Metselaar, Eduarda Fernandes, Pedro V. Baptista, Alexandra R. Fernandes, Maria Luísa Corvo

**Affiliations:** 1 Faculty of Pharmacy, iMed.ULisboa–Research Institute for Medicines, University of Lisbon, Av. Prof. Gama Pinto, 1649-003 Lisbon, Portugal; ana.m.silva@campus.ul.pt (M.F.-S.); ana.catarina.silva@campus.ul.pt (C.F.-S.); manuela.colla@campus.ul.pt (M.C.C.); ssimoes@ff.ulisboa.pt (S.S.); 2 Associate Laboratory i4HB, Campus da Caparica, Institute for Health and Bioeconomy, NOVA School of Science and Technology, NOVA University Lisbon, 2819-516 Caparica, Portugal; pmvb@fct.unl.pt; 3 UCIBIO–Applied Molecular Biosciences Unit, Department of Life Sciences, Campus da Caparica, NOVA School of Science and Technology, NOVA University Lisbon, 2819-516 Caparica, Portugal; 4 LAQV, REQUIMTE–Laboratory of Applied Chemistry, Department of Chemical Sciences, Faculty of Pharmacy, University of Porto, Rua de Jorge Viterbo Ferreira, 228, 4050-313 Porto, Portugal; egracas@ff.up.pt; 5 CQB–Centro de Química Estrutural, DQB–Departamento de Química e Bioquímica, Faculdade de Ciências, Universidade de Lisboa, 1749-016 Lisboa, Portugal; hsmarinho@fc.ul.pt; 6 CEDOC, Campo dos Mártires da Pátria 130, Nova Medical School, 1169-056 Lisboa, Portugal; p.marcelino@netcabo.pt; 7 ANATOMIK–Laboratório de Anatomia Patológica, Grupo Clara Saúde, Rua Gago Coutinho e Sacadura Cabral, 136–138, 2955-190 Palmela, Portugal; celestecamposstar@gmail.com; 8 Institute for Experimental Molecular Imaging, RWTH Aachen University Clinic, 52074 Aachen, Germany; jmetselaar@ukaachen.de

**Keywords:** hepatic ischemia and reperfusion injury, liposomes, quercetin, inflammation, anti-inflammatory therapy, drug delivery nanosystems, liver

## Abstract

Ischemia and reperfusion injury (IRI) is a common complication caused by inflammation and oxidative stress resulting from liver surgery. Current therapeutic strategies do not present the desirable efficacy, and severe side effects can occur. To overcome these drawbacks, new therapeutic alternatives are necessary. Drug delivery nanosystems have been explored due to their capacity to improve the therapeutic index of conventional drugs. Within nanocarriers, liposomes are one of the most successful, with several formulations currently in the market. As improved therapeutic outcomes have been demonstrated by using liposomes as drug carriers, this nanosystem was used to deliver quercetin, a flavonoid with anti-inflammatory and antioxidant properties, in hepatic IRI treatment. In the present work, a stable quercetin liposomal formulation was developed and characterized. Additionally, an in vitro model of ischemia and reperfusion was developed with a hypoxia chamber, where the anti-inflammatory potential of liposomal quercetin was evaluated, revealing the downregulation of pro-inflammatory markers. The anti-inflammatory effect of quercetin liposomes was also assessed in vivo in a rat model of hepatic IRI, in which a decrease in inflammation markers and enhanced recovery were observed. These results demonstrate that quercetin liposomes may provide a significant tool for addressing the current bottlenecks in hepatic IRI treatment.

## 1. Introduction

Ischemia and reperfusion injury (IRI) is a common complication, capable of hindering the success of the growing number of liver surgeries [1] due to the development of oxidative stress and inflammation [2,3]. The current therapy for liver IRI includes non-steroid anti-inflammatory drugs (NSAID) and corticosteroids that do not achieve the desirable efficacy and additionally can present severe side effects [4]. Therefore, novel and more effective therapeutic approaches are needed.

The use of drug delivery nanosystems (DDS), known to enhance the therapeutic index, reduce the required drug load, and minimize adverse side effects [5,6,7], could be beneficial in overcoming the hindrances presented by current IRI treatments. In this context, liposomes are one of the most promising nanocarriers, with several formulations approved for clinical use [8]. In addition to the possibility of tailoring the characteristics of liposomal formulations according to the desired purpose (e.g., to cross biological barriers or accumulate in specific sites), they also offer other advantages including biocompatibility, biodegradability, low toxicity, capacity to transport large quantities of therapeutic agents, and ability to self-assemble [9,10].

Quercetin is a plant-derived flavonoid whose intake was estimated to range from 37.5 to 600 mg/day (depending on the consumption of fruit, vegetables, and tea) [11]. Anti-inflammatory and antioxidant properties have been attributed to this compound due to its ability to increase production of anti-inflammatory cytokines, to reduce the production of pro-inflammatory cytokines and reactive oxygen species (ROS), and to prevent lipid peroxidation [12,13,14]. In the majority of the studies, this flavonoid is administered as a dietary supplement. However, quercetin presents poor water solubility and low bioavailability [15], which greatly restricts its therapeutic potential.

In the present work, we focused on tackling this bottleneck via the incorporation of quercetin in a long-circulating liposomal formulation, which was evaluated as an alternative therapeutic approach. The nanoformulation was characterized and studied in an in vitro model of ischemia and reperfusion (IR) and in an in vivo model of hepatic IRI to evaluate the therapeutic potential of this quercetin liposomal formulation in hepatic IRI.

## 2. Materials and Methods

### 2.1. Preparation of Quercetin Liposomes

Quercetin nanoformulations were prepared by the thin-film hydration method [16], followed by sequential extrusion. Briefly, quercetin (>97%, Alfa Aesar, Ward Hill, MA, USA) was dissolved in methanol (HPLC gradient grade, Merck, Darmstadt, Germany), and this solution was added to the lipid components (cholesterol (Chol—Merck, Darmstadt, Germany), soybean L-α-phosphatidylcholine (SPC—Lipoid S100, Lipoid GmbH, Ludwigshafen, Germany), and 1,2-distearoyl-sn-glycero-3-phosphoethanolamine-N-[methoxy(polyethylene glycol)-2000] (DSPE-mPEG_2000_—Lipoid GmbH, Germany)) that were dissolved in chloroform (≥99.9%, Merck, Germany) and then evaporated until dry in a rotatory evaporator (Buchi Rotavapor RE-111, Lugo, Switzerland), forming a lipid film. The film was hydrated with citrate buffer (10 mM citrate buffer, 145 mM NaCl, pH 6.0), stirred, and rested for 1 h to form multi-lamellar vesicles. These vesicles were sized by sequential extrusion through PTFE membrane filters with pore size ranging from 800 to 100 nm in a Lipex™ Thermobarrel Extruder (TRANSFERRA Nanosciences Inc., Burnaby, BC, Canada) under nitrogen pressure (10–500 lb/in^2^). Finally, the formulation was purified by size exclusion chromatography using a desalting column with a 1000 Dalton cutoff (Econo-Pac^®^, BIO-RAD, Hercules, CA, USA), concentrated by ultracentrifugation at 300,000× *g*, 2 h, at 15 °C in an Optima™ XL-90 Ultracentrifuge (Beckman Coulter, Carlsbad, CA, USA) and filtered through syringe filters PTFE 0.2 µm.

### 2.2. Characterization of Nanoformulations

Liposomal formulations were characterized in citrate buffer (10 mM citrate buffer, 145 mM NaCl, pH 6.0) according to their mean hydrodynamic diameter, polydispersity index in a Zetasizer Nano S (Malvern Instruments, Malvern, UK), and surface charge in a Zetasizer Nano Z (Malvern Instruments, Malvern, UK). The absence of quercetin crystals was evaluated by microscopy over time, and the lipid quantification was performed by the Rouser method [17].

Drug quantification was performed by high-performance liquid chromatography (HPLC) in a Purospher^®^ STAR RP-18 endcapped (5 µm) HPLC column, 4.6 × 250 mm (Merck, Darmstadt, Germany), using a Beckman System Gold HPLC (Beckman Coulter, Carlsbad, CA, USA). Liposomes containing drug were disrupted with methanol, with the concomitant solubilization of quercetin, and filtered through a PTFE membrane with 0.2 µm pore diameter before quantification at 360 nm with a mobile phase consisting of methanol/water acidified with 1 mM trifluoroacetic acid (70:30, *v*/*v*).

Nanoformulations were evaluated in terms of loading capacity, defined as the final drug-to-lipid ratio (µg quercetin/µmol lipid)–([Drug]/[Lipid])f–, and the incorporation efficiency (I.E.) that was determined according to the following equation:

(1) $$I.E.=\frac{\left(\frac{\left[\mathrm{Drug}\right]}{\left[\mathrm{Lipid}\right]}\right)f}{\left(\frac{\left[\mathrm{Drug}\right]}{\left[\mathrm{Lipid}\right]}\right)i}\times 100$$

### 2.3. Stability Studies

Liposomes stability on storage was evaluated in freshly prepared liposomal suspensions under different conditions: at 4 °C for 90 days and at 37 °C for 48 h, in citrate buffer (10 mM citrate buffer, 145 mM NaCl, pH 6.0) with or without 1% (*w*/*v*) bovine serum albumin (BSA—Merck, Darmstadt, Germany). Aliquots were collected, non-encapsulated drug was removed by size exclusion chromatography using a desalting column of 1000 Dalton cutoff (Econo-Pac^®^, BIO-RAD, Hercules, CA, USA), and a complete characterization of stable liposomes was repeated.

### 2.4. Release Studies

Drug released from the nanosystems was evaluated at different time points over a period of 24 h at 37 °C, using a Franz diffusion cell system with a cellulose dialysis membrane of 12–14 kDa cutoff between the donor and receptor phase. The receptor phase was a solution of 1% (*w*/*v*) BSA in phosphate-buffered saline (PBS, Merck, Darmstadt, Germany), pH 7.4. In the donor compartment, formulation was diluted with PBS with or without 1% (*w*/*v*) BSA.

### 2.5. Hypoxia Chamber as In Vitro Model of Ischemia and Reperfusion

Hepatocellular carcinoma (HepG2) cells (ATCC^®^, Manassas, VA, USA) were seeded in complete medium—DMEM (Gibco™, Darmstadt, Germany) supplemented with 10% (*v*/*v*) fetal bovine serum (FBS—ATCC^®^, Manassas, VA, USA) and 1% (*v*/*v*) antibiotic–antimycotic solution (Merck, Darmstadt, Germany)—into a 96-well plate at 0.75 × 10^5^ cells/mL for MTS assays; into a 24-well plate at 2.5 × 10^5^ cells/mL for RNA extraction; or at 1.0 × 10^5^ cells/mL for ROS production assays. After 48 h, medium was replaced by fresh DMEM (supplemented or not with FBS) containing 10 µM or 500 µM quercetin liposomes or free drug (solubilized in 0.1% dimethyl sulfoxide—DMSO, ≥99.9%, Merck, Darmstadt, Germany). Immediately or 6 h after the incubation with liposomes, cells were placed in the hypoxia chamber BD BBL™ GasPak™ jar (Becton, Dickinson and Company, Franklin Lakes, NJ, USA) with an anaerobic generator (bioMérieux SA, Craponne, France) and anaerobic indicator (bioMérieux SA, Craponne, France), at 37 °C. After 10 h, medium was removed, and cells incubated for an additional 4 h with fresh complete DMEM in an incubator at 37 °C with a humidified atmosphere of 5% CO_2_.

For the HUVEC cell line (ATCC^®^, Manassas, VA, USA), a cell density of 1.5 × 10^5^ cells/mL was seeded for MTS assays or 5 × 10^5^ cells/mL for RNA extraction assays. The assay was performed using Kaighn’s Modification of Ham’s F-12 Medium (F-12K—ATCC^®^, Manassas, VA, USA), supplemented with 10% (*v*/*v*) FBS, 1% (*v*/*v*) endothelial cell growth supplement from bovine neural tissue (Merck, Darmstadt, Germany), and 0.1 mg/mL heparin sodium salt from porcine intestinal mucosa (Merck, Darmstadt, Germany)—F-12K complete medium. For co-culture of HepG2/HUVEC at 2:1 ratio, the same cell density previously described for HepG2 was used, with F-12K complete medium.

Controls with unloaded liposomes or without liposomes were also performed. A similar procedure was applied in normoxia conditions, where cells were placed 10 h in the incubator instead of the hypoxia chamber.

### 2.6. Cellular Viability Assay

For MTS assays, after the protocol described in the hypoxia chamber model, medium was removed and replaced with a solution composed of complete medium and MTS (CellTiter 96^®^ Aq_ueous_ One Solution Cell Proliferation Assay, Promega, Madison, WI, USA) at a 4:1 ratio (*v*:*v*), followed by 45 min incubation at 37 °C. After that period, absorbance was measured at 490 nm in a microplate reader Infinite^®^ 200 (Tecan Trading AG, Männedorf, Switzerland). Appropriate blank controls were included in each assay. Cell proliferation analysis was carried out in GraphPad Prism^®^7 (GraphPad Software, San Diego, CA, USA).

### 2.7. RNA Extraction

For RNA extraction assays, after the protocol described in the hypoxia chamber model, medium was removed, and cells were lysed with 300 µL of NZYol (NZYtech, Lisbon, Portugal). After 5 min incubation at room temperature, 150 µL of chloroform (≥99.9%, Merck, Darmstadt, Germany) was added and was vigorously shaken. Afterwards, a centrifugation at 12,000× *g* for 5 min was performed, and the RNA from the upper aqueous phase was precipitated by adding 500 µL of isopropanol (≥99.5%, Merck, Darmstadt, Germany). After a 10 min incubation at room temperature, the solution was centrifuged at 12,000× *g* for 10 min at 4 °C. The pellet was resuspended in 500 µL of 75% (*v*/*v*) ethanol (≥99.8%, Merck, Darmstadt, Germany) and centrifuged at 7500× *g* for 5 min at 4 °C. The precipitated RNA was air dried for 5–10 min, resuspended in DEPC-treated water, and incubated in a heat block at 60 °C for 10 min. The quality and concentration of isolated RNA was assessed using a NanoDrop^®^ ND-1000 spectrophotometer (Thermo Fisher Scientific, Waltham, MA, USA). RNA integrity of each sample was evaluated by denaturing agarose gel electrophoresis (0.8% (*w*/*v*) agarose), and samples were stored at −80 °C. Gel images were captured using the Gel Doc XR system and Quantity One 1-D analysis software (Bio-Rad Laboratories, Hercules, CA, USA).

### 2.8. RNA Purification

For RNA purification, 1 µg RNA was incubated with 1 µL NZY DNase I (NZYtech, Lisbon, Portugal) at 37 °C for 20 min. Then, 200 µL absolute ethanol (Merck, Darmstadt, Germany) was added, and samples were stored at −20 °C overnight. On the following day, a centrifugation at 15,000× *g* for 15 min at 4 °C was performed, precipitated RNA was suspended in 100 µL of 75% (*v*/*v*) ethanol, and further centrifuged at 15,000× *g* for 15 min at 4 °C. The pellet was air dried and afterwards was hydrated with DEPC-treated water and incubated in a heat block at 60 °C for 10 min. The quality and concentration of isolated RNA was assessed using a NanoDrop^®^ ND-1000 spectrophotometer (Thermo Fisher Scientific, Waltham, MA, USA), and purified RNA was stored at −80 °C.

### 2.9. cDNA Synthesis

The cDNA was synthesized from 100 ng of RNA using an NZY M-MuLV First-Strand cDNA Synthesis Kit (NZYtech, Lisbon, Portugal) for a final volume of 10 µL. Samples were incubated in DNA Engine^®^ Thermal Cycler (Bio-Rad Laboratories, Hercules, CA, USA) at 25 °C for 10 min, 37 °C for 50 min, and 85 °C for 5 min. Afterwards, 0.5 µL of RNase H (NZYtech, Lisbon, Portugal) was added and samples were stirred in a vortex and incubated in a heat block at 37 °C for 20 min. The synthetized cDNA was stored at −20 °C for further analysis.

### 2.10. RT-qPCR

The inflammatory markers gene expression was analyzed by quantitative reverse transcription polymerase chain reaction (RT-qPCR) using the synthetized cDNA as a template. The 18S ribosomal RNA (*18S*) gene was used as an endogenous control. Relative gene expression levels were quantified based on the threshold cycle (2^−ΔΔCT^) method [18], where:

ΔΔCT = (CT_inflammatory marker_ − CT_18S_)_sample_ − (CT_inflammatory marker_ − CT_18S_)_calibrator_ (2)

was used to analyze gene expression obtained using the hypoxia chamber model, the reaction mixture for RT-qPCR was prepared according to the manufacturer’s instructions, and RT-qPCR was performed in a Rotor-Gene™ 6000 (Corbett Life Science, Cambridge, UK). In the case of tumor necrosis factor α (TNF-α, gene: *TNFA*) and interleukin 6 (IL-6, gene: *IL6*), for a final volume of 10 μL the mixture included 5 μL TaqMan^®^ Fast Advanced Master Mix (Applied Biosystems, Waltham, MA, USA), 1 μL cDNA, and 0.5 μL TaqMan^®^ Gene Expression Assays for *18S*, *TNFA*, and *IL6* (Hs03003631_g1, Hs00174128_m1, Hs00174131_m1, respectively—Applied Biosystems, Waltham, MA, USA). The conditions included an initial incubation at 50 °C for 2 min, followed by an incubation at 95 °C for 1 min for enzyme activation, followed by 40 cycles of amplification consisting of denaturation at 95 °C for 5 s and annealing and extension at 60 °C for 30 s. For hypoxia-inducible factor 1 α (HIF-1 α, gene: *HIF1A*), the 20 μL reaction mixture was composed of 10 μL NZY qPCR Green Master Mix (NZYtech, Lisbon, Portugal), 2 μL cDNA, and 0.2 μM of forward and reverse gene-specific primers for *18S* and *HIF1A* (STAB VIDA, Setúbal, Portugal), depicted in Table 1. The conditions included an initial denaturation at 95 °C for 2 min, followed by 10 cycles of amplification consisting of denaturation at 95 °C for 20 s, annealing at 50 °C for 10 s, and extension at 72 °C for 20 s and by 30 cycles amplification consisting of denaturation at 95 °C for 20 s, annealing at 58 °C for 10 s, and extension at 72 °C for 20 s. For interleukin 10 (IL-10, gene: *IL10*), the 10 μL reaction mixture was composed of 2 μL HOT FIREPol^®^ EvaGreen^®^ qPCR Mix Plus (Solis BioDyne, Tartu, Estonia), 1 μL cDNA, and 0.2 μM of forward and reverse gene-specific primers for *18S* and *IL10* (STAB VIDA, Setúbal, Portugal) indicated in Table 1. The conditions included an initial denaturation at 95 °C for 15 min, followed by 10 cycles of amplification consisting of denaturation at 95 °C for 20 s, annealing at 50 °C for 20 s, and extension at 72 °C for 20 s and by 35 cycles amplification consisting of denaturation at 95 °C for 20 s, annealing at 53 °C for 20 s, and extension at 72 °C for 20 s and a final extension step at 72 °C for 10 min.

### 2.11. ROS Production

For ROS production assays, a modification was included in the protocol described in the hypoxia chamber model—1 μM CellROX™ Green Reagent (Invitrogen, Carlsbad, CA, USA) was added simultaneously with the treatment and at the reperfusion onset. After the incubation, the culture medium was removed, cells were washed twice with PBS, and were fixed with a 4% (*w*/*v*) paraformaldehyde solution (Merck, Germany). The emission of green fluorescence was observed in a Ti-U Eclipse inverted fluorescence microscope (Nikon, Tokyo, Japan) with a FITC fluorescence filter cube with an excitation filter of 465–495 nm, a dichroic mirror at 505 nm, and a barrier filter at 515–555 nm (Nikon, Tokyo, Japan). Images were captured using NIS Elements Basic software (Nikon, Tokyo, Japan) and analyzed with ImageJ software v1.53k, where the corrected total cell fluorescence was calculated by subtracting the background fluorescence to the intensity of cell fluorescence measured. To control fluorescence intensity, acquisition times were the same in all fluorescence images, and values were expressed as fluorescence ratio (hypoxia versus normoxia). An amount of 100 μM tert-butyl hydroperoxide (TBHP—Luperox^®^ TBH70X, Merck, Darmstadt, Germany) was used as a positive control.

### 2.12. Animal Model and Surgical Procedure

Male Wistar Han rats aged 56 to 62 days were purchased from Charles River, Saint Germain Nuelles, France. Animals were kept under standard hygiene conditions, fed commercial chow, and given acidified drinking water ad libitum. All animal experiments were conducted according to the animal welfare organ of the Faculty of Pharmacy, University Lisbon, approved by the competent national authority Direção-Geral de Alimentação e Veterinária, Lisbon, Portugal (DGAV), and in accordance with the Declaration of Helsinki, Portuguese laws (DR 113/2013, 2880/2015, 260/2016 and 673/2018), and with the EU Directive (2010/63/UE) and its amendment (Regulation (EU) 2019/1010).

Rats were randomly divided in groups (4 animals per group), and quercetin nanoformulation (SPC_Querc) was administered at 1.3 mg/kg body weight by intravenous (i.v.) route 24 h before the surgical procedure. In naive and anesthesia control groups, animals were not subjected to surgery, but in the latter, anesthesia and its reversing agent were injected by intramuscular (i.m.) route as occurs when the surgery is performed. A sham control group was also included, i.e., the surgery was performed but no formulation was administered (IR group).

Animals were weighted and anesthetized with an i.m. dose of 75 mg/kg ketamine (Imalgene 1000, Merial, Duluth, GA, USA) combined with 1 mg/kg medetomidine (Domitor^®^, Orion Corporation, Turku, Finland).

After anesthetic induction, a trichotomy was performed in the abdominal area, and a midline laparotomy was executed to expose the liver hilum. The portal vein and hepatic artery were clamped with a vascular microclamp (Dieffenbach Vessel Clip, Straight, 35.0 mm, Harvard Apparatus, Holliston, MA, USA), interrupting the blood supply to the three cephalic lobes for 20 min, and body temperature was maintained with an electronic heating pad. Hepatic ischemia was verified visually by the change in the liver coloration into a paler shade. After the removal of the clamp to restore the liver blood flow and the observation of the regular liver coloration, laparotomy wounds were sutured, and 1 mg/kg atipamezole (Sedastop^®^, B. Braun, Bethlehem, PA, USA) was administered by i.m. route for anesthesia reversal. Animals were monitored for pain or distress, and body weight was registered before sacrifice. At two distinct time points (6 h and 24 h) post-surgery, blood was collected under isoflurane (IsoVet, B.Braun, Bethlehem, PA, USA) anesthesia by cardiac puncture for biochemical parameters evaluation. Afterwards, animals were euthanized with an isoflurane overdose and death was confirmed by cervical dislocation. The liver was excised, washed with a 0.154 mM potassium chloride solution (Merck, Darmstadt, Germany), weighed, and divided according to the desired purpose: a portion of left lobe was placed in RNAlater™ stabilization solution (Invitrogen, Carlsbad, CA, USA) and stored at 4 °C for determination of mRNA pro-inflammatory biomarker expression level, and the right lobe was placed in a 10% (*v*/*v*) neutral buffered formalin solution (Merck, Darmstadt, Germany) for histological and histochemical analysis.

### 2.13. mRNA Expression Analysis from Liver

RNA was extracted from liver samples with SV Total RNA Isolation System (Promega, Madison, WI, USA), as described by the manufacturer for mammalian tissues, cDNA was synthetized as previously mentioned, and *TNF* mRNA expression level was assessed by RT-qPCR in a final volume of 10 μL that contained 5 µL Green Enzyme Mix (NZYtech, Lisbon, Portugal, 1 µL cDNA, and 0.1 µM forward and reverse gene-specific primers (STAB VIDA, Setúbal, Portugal) depicted in Table 2.

RT-qPCR was performed in a Rotor-Gene™ 6000 (Corbett Life Science, Cambridge, UK), and conditions included an initial denaturation at 95 °C for 5 min, followed by 35 cycles amplification consisting of denaturation at 95 °C for 30 s, annealing at 62 °C for 10 s, and extension at 72 °C for 15 s and a final extension step at 72 °C for 10 min. 

PCR products were analyzed by electrophoresis in 2% (*w*/*v*) agarose gel using the Gel Doc XR system and Quantity One 1-D analysis software (Bio-Rad Laboratories, Hercules, CA, USA). The molecular weight marker GeneRuler 50 bp DNA Ladder (Thermo Fisher Scientific, Waltham, MA, USA) was used to estimate PCR products size obtained.

### 2.14. Biochemical, Histological, and Histochemical Analysis

Hepatic biochemical parameters—namely, serum aspartate transaminase (AST), alanine transaminase (ALT), alkaline phosphatase (ALP) and γ-glutamyltransferase (GGT) activity levels—were determined by DNAtech (Lisbon, Portugal), and liver histological and histochemical analysis was performed by ANATOMIK (Palmela, Portugal).

### 2.15. Statistical Analysis

Results are expressed as mean ± standard deviation (SD). Statistical analysis was performed using two-way ANOVA followed by Tukey’s multiple comparisons test using GraphPad Prism^®^7 (GraphPad Software, San Diego, CA, USA). A value of *p* < 0.05 was considered statistically significant.

## 3. Results and Discussion

### 3.1. Liposomal Formulation Characterization

**Figure 1:**
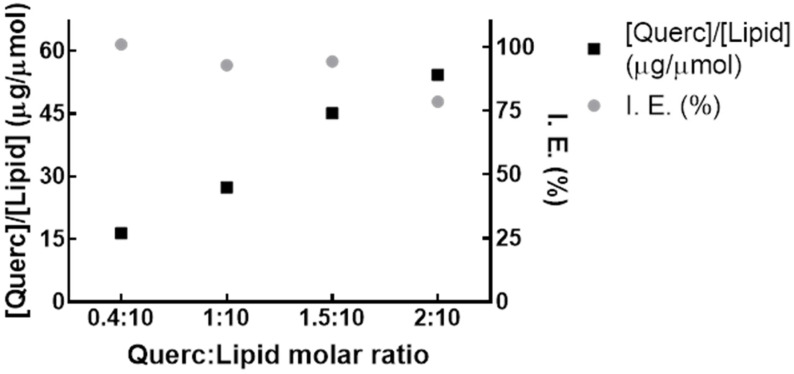
Saturation profile of quercetin liposomes. Saturation curves obtained through the initial molar ratio between quercetin (Querc) and lipid concentrations ([Querc]/[Lipid]) and quercetin incorporation efficiency (I.E.) (*n* = 2).

Quercetin nanoformulations were developed with distinct drug-to-lipid molar ratios to obtain a quercetin saturation profile for evaluating the most suitable composition. From the saturation curve obtained, observed in Figure 1, a quercetin-to-lipid molar ratio of 1:10 (mol quercetin/mol lipid) was selected since the incorporation efficiency obtained was around 100%, being superior to the values reported in literature [19,20,21]. Moreover, at drug-to-lipid molar ratios above 1:10, precipitation events occurred during extrusion process.

Liposomal formulations were fully characterized according to their mean diameter, superficial charge, loading capacity, and incorporation efficiency (I.E.). The selected formulation presented a mean size of around 0.12 µm with low polydispersity index (PDI ˂ 0.1), neutral superficial charge, and a loading capacity of 31 ± 3 µg quercetin/µmol lipid (or 0.09 ± 0.01 µmol quercetin/µmol lipid), as observed in Table 3.

**Figure 2:**
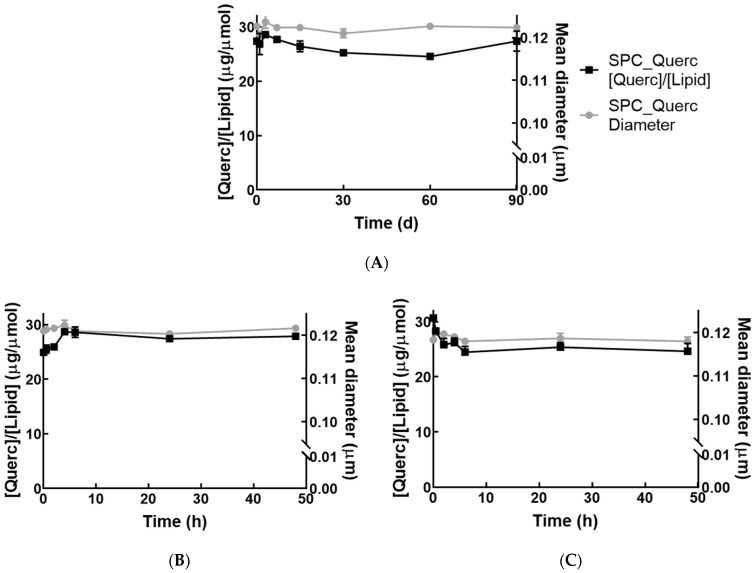
Stability of quercetin liposomes. Stability of SPC_Querc at 4 °C in citrate buffer (**A**) or at 37 °C in citrate buffer without (**B**) or with 1% (*w*/*v*) BSA (**C**). Values of the loading capacity (quercetin-to-lipid ratio, [Querc]/[Lipid]) and mean diameter are represented as mean ± SD (*n* = 3).

Liposomal quercetin stability was evaluated after the removal of non-incorporated drug in two distinct settings: 3 months at 4 °C in citrate buffer (Figure 2A) to assess the storage stability and 2 days at 37 °C in citrate buffer without or with 1% (*w*/*v*) BSA (Figure 2B,C, respectively). BSA was used to improve the in vitro–in vivo drug release correlation since albumin is adsorbed by liposomes mimicking more closely the physiological conditions [22]. In all settings, it was possible to observe that both quercetin-to-lipid ratio and mean diameter remained constant. The loading capacity—measured as drug-to-lipid ratio (µg quercetin/µmol lipid)—and the mean diameter obtained during the study were equal to that measured after liposomes preparation (31 ± 2 µg Querc/µmol Lipid and 0.12 ± 0.01 µm—Table 3). This indicated that the developed liposomes were stable even in the presence of BSA for 48 h at 37 °C. Quercetin liposomes stability is of the utmost importance because it will enable their hepatic accumulation in inflammatory sites due to the enhanced permeability and retention (EPR) effect [23].

**Figure 3:**
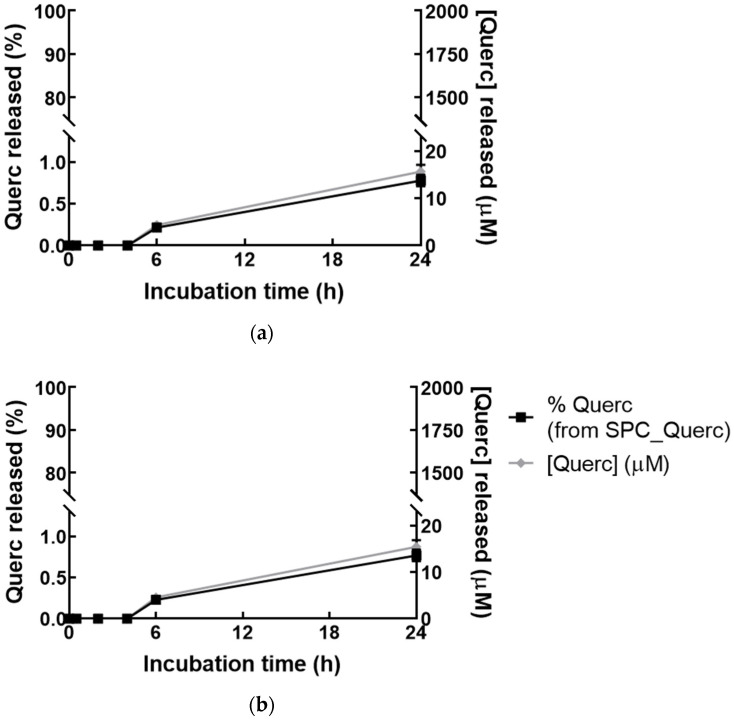
Quercetin release from liposomal formulation. Percentage of quercetin (Querc) released from the initially incorporated drug (%) in SPC_Querc formulation and the correspondent quercetin concentration ([Querc]) released in PBS with 1% (*w*/*v*) BSA (**a**) or PBS (**b**), during 24 h. Values represented as mean ± SD (*n* = 3).

To further improve the in vivo predictions, liposomal quercetin release studies were performed. The release profiles are presented in Figure 3, and it could be observed that, up to 4 h, no quercetin release was detected in the receptor compartment, and that after 24 h, less than 1.5% of quercetin (or 14 µM) was released from the formulation, even in the presence of 1% (*w*/*v*) BSA. This result confirms that minimal drug leakage is expected to occur 24 h after formulation administration. This characteristic is crucial for a successful therapy in hepatic IRI since liposomes will accumulate over time in the inflamed liver due to EPR effect, and minimal drug release will maximize their therapeutic effect. Moreover, the occurrence of off-targeted side effects will also decrease since the drug will be released mostly at the intended site of action. The maximization of therapeutic effect and minimization of side effect are clear advantages in therapy, where long-circulating liposomes are more beneficial for hepatic IRI treatment than free quercetin. Altogether, the stability studies demonstrated that quercetin liposomes presented suitable characteristics for the evaluation of their in vitro potential as an alternative therapy.

### 3.2. In Vitro Studies

**Figure 4:**
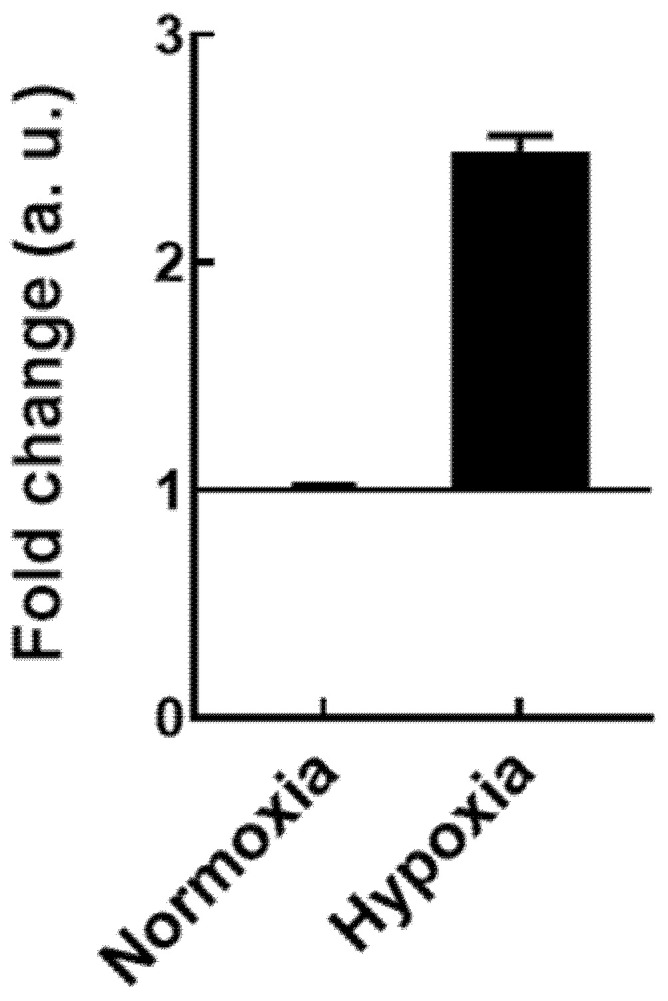
HIF-1α mRNA expression level obtained in normoxia or hypoxia conditions in HepG2 cells. Results were normalized to cells in normoxia and are presented as median ± SD (*n* = 4).

The induction of hypoxia in cells during the 10 h incubation period inside the hypoxia chamber was confirmed through the mRNA expression of HIF-1α—a transcription factor reported as upregulated upon hypoxia [24,25,26,27]. A 2.5-fold overexpression of HIF-1α was obtained (Figure 4) in HepG2 cells that incubated 10 h inside the hypoxia chamber in comparison with cells that were placed in the incubator during the same incubation period, confirming that the developed in vitro IR model was effectively inducing hypoxia in cells.

In the developed IR in vitro model, during hypoxia period, HepG2 cells were incubated with complete medium or with DMEM without any supplement (simple medium). Additionally, SPC_Querc were also added 6 h before or at the onset of hypoxia to investigate their effect.

**Figure 5:**
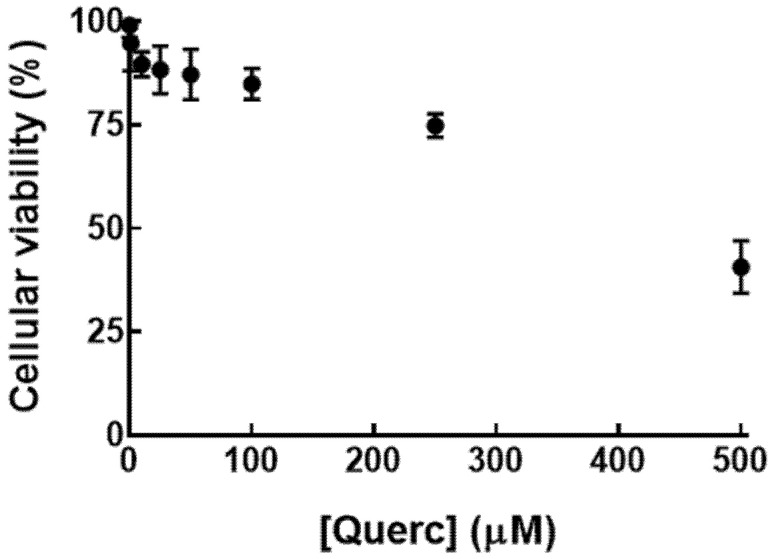
HepG2 cells viability after incubation with free quercetin for 24 h. Viability was determined with a quercetin concentration ([Querc]) ranging from 0 to 500 µM. Results presented as mean ± SD (*n* = 3).

HepG2 cells viability was evaluated within a range from 1 to 500 µM free quercetin to select the appropriate concentrations for the subsequent studies (Figure 5).

**Figure 6:**
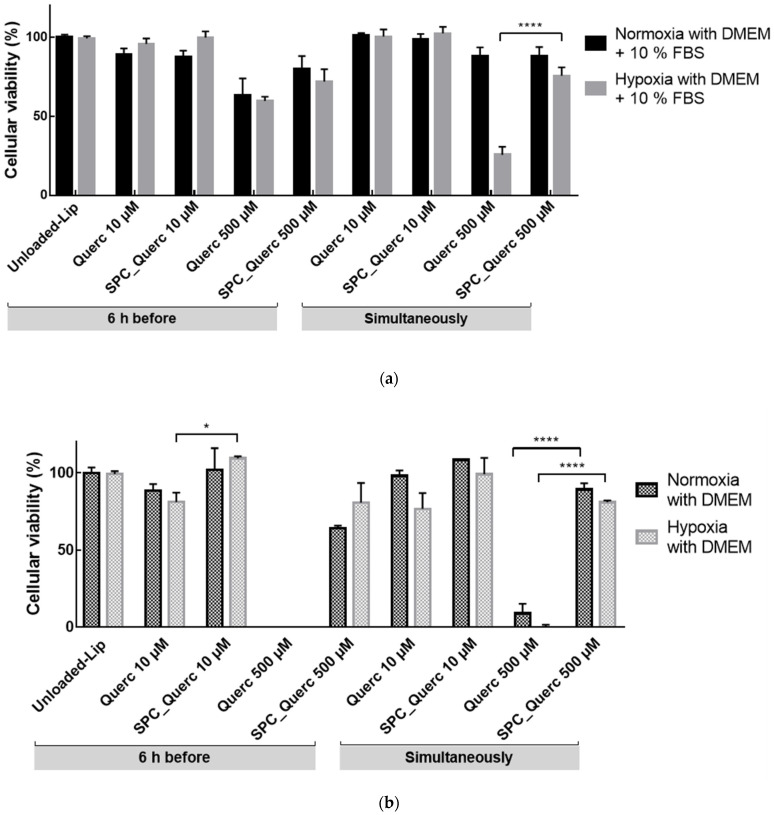
HepG2 cells viability upon incubation with 10 or 500 µM quercetin. Viability was evaluated either with liposomal or free quercetin, under hypoxia or normoxia conditions, in the presence of complete (**a**) or simple (**b**) medium. Unloaded-liposomes (Unloaded-Lip) were used as control. Results presented as mean ± SD (*n* = 3). * *p* < 0.05 and **** *p* < 0.0001.

HepG2 cells viability was maintained above 90% upon 24 h incubation with free quercetin concentration up to 50 µM. For the subsequent studies, a concentration of 10 µM and 500 µM, representing 97% and 40% of viability, respectively, were applied to evaluate the effect of hypoxia in HepG2 cells. From Figure 6 it is possible to observe that, at the lower concentration, both free and liposomal forms were able to maintain HepG2 cells viability above 87%, even when incubated with simple medium during hypoxia. The opposite effect was observed with 500 µM free quercetin, where cells viability was reduced to 33% when incubated with complete DMEM and 1% with simple DMEM. Liposomes were able to attenuate that toxicity, leading to a minimum viability of 64% (Figure 5). This unveils a protective effect of this drug delivery system, demonstrating its potential in decreasing in vitro drugs toxicity, as observed for dexamethasone [28].

**Figure 7:**
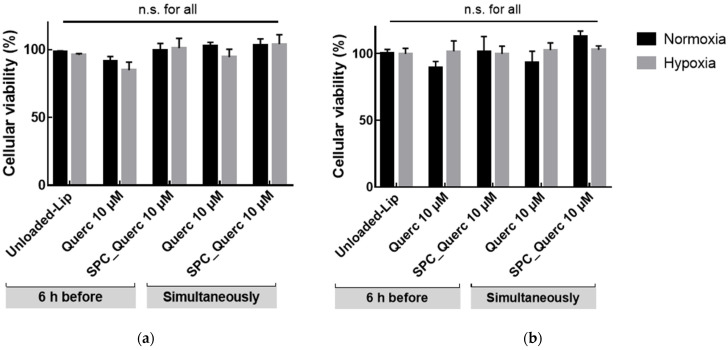
HUVEC (**a**) or HepG2:HUVEC (**b**) cells viability upon incubation with 10 µM quercetin. Viability was evaluated either with liposomal or free quercetin, under hypoxia or normoxia conditions, in the presence of complete medium. Unloaded-liposomes (Unloaded-Lip) were used as control. Results presented as mean ± SD (*n* = 3). n.s., statistically non-significant (free vs. liposomal quercetin, at the same time of addition).

As a strategy for improving the in vitro predictions of the formulation effect in liver IRI, the effect of quercetin was evaluated in a more complex in vitro model that resembles more the in vivo situation. As liver is composed by hepatocytes—that constitute around 70% of liver and present metabolic and detoxification functions—and non-parenchymal cells—namely, endothelial cells, Kupffer cells, lymphocytes, stellate cells, and biliary cells [29,30]—HUVEC were used as a representative type of endothelial cells [31], alone or in co-culture with HepG2 cells within this model (Figure 7).

Cell viability was initially assessed in a HUVEC mono-culture model. In this model, cell viability was maintained under normoxia and hypoxia conditions when incubated with 10 µM quercetin nanoformulation in complete medium, as depicted in Figure 7a. Afterwards, a HepG2/HUVEC co-culture model was also studied [32,33]. Data obtained for HepG2/HUVEC co-cultures revealed that cell viability was also maintained under hypoxia with complete medium with 10 µM quercetin nanoformulation, as depicted in Figure 7b.

**Figure 8:**
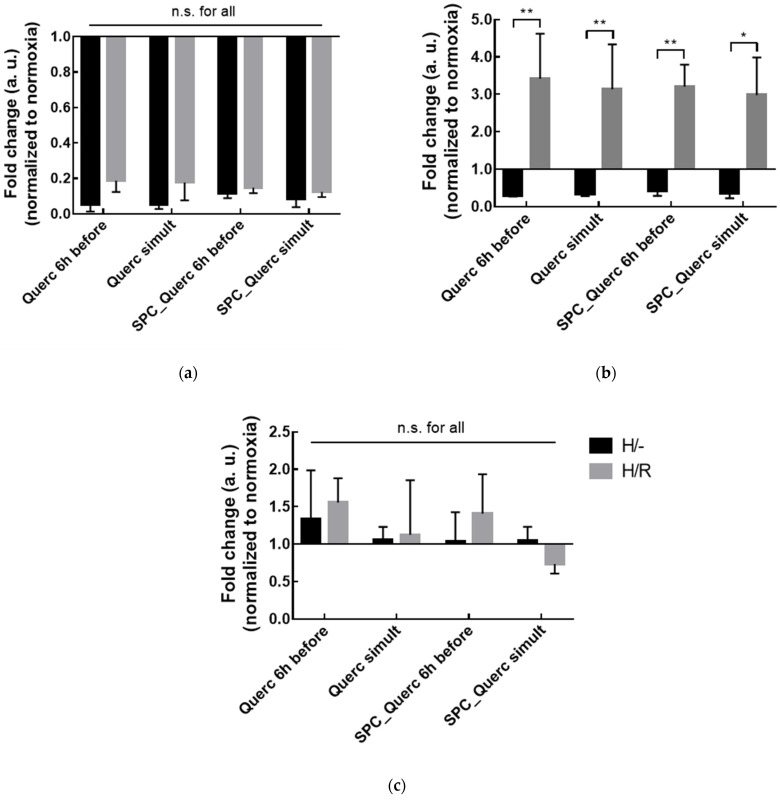
Fold change in TNF-α (**a**), IL-6 (**b**), or IL-10 (**c**) levels in HepG2 cells incubated with 10 µM of free quercetin (Querc) or quercetin liposomes (SPC_Querc) added 6 h before or simultaneously with the hypoxia onset (simult). The biomarkers were analyzed at the end of the hypoxia period (H- or after the 4 h reperfusion period (H/R). Results presented as mean ± SD of the fold change, where each condition was normalized to vehicle control in normoxia—cells incubated with 0.1% DMSO or unloaded-liposomes were used as Querc or SPC_Querc controls, respectively (*n* = 4). * *p* < 0.05 and ** *p* < 0.01; n.s., statistically non-significant (H/- vs. H/R and free vs. liposomal quercetin in the same condition).

The non-toxic concentration of 10 µM quercetin was chosen to perform the subsequent studies, where the expression of inflammatory markers from cells treated with free or liposomal quercetin and incubated 10 h in hypoxia was compared with the negative controls (cells maintained in normoxia and without any treatment) (Figure 8).

In Figure 8, the downregulation of the pro-inflammatory markers could be observed when cells were incubated with free or liposomal quercetin added 6 h before or simultaneously with hypoxia onset. In the case of TNF-α, the decreased expression occurred either in the absence or presence of the reperfusion period, but for IL-6, the downregulation was only observed in the absence of reperfusion. From these results, quercetin treatment demonstrated a higher anti-inflammatory effect during the hypoxia period, where it was capable to decrease both biomarkers’ expression. Despite the reversion of the anti-inflammatory effect for IL-6 after the 4 h reperfusion period where the drug was not present, quercetin was able to maintain this anti-inflammatory effect for TNF-α, indicating that free or liposomal quercetin potentially displays a prolonged impact in inflammation even after their removal. Differences in the expression of the anti-inflammatory marker IL-10 were less accentuated than the obtained for the pro-inflammatory markers, possibly due to the different profiles of inflammatory response—initially the pro-inflammatory response is triggered and only afterwards is the anti-inflammatory response developed [34]. Since in this study all the biomarkers were evaluated at the same time point, IL-10 mRNA expression was possibly detected in the initial phase of the anti-inflammatory response. Nonetheless, a slight increase in IL-10 expression was observed, mainly when cells were treated 6 h before hypoxia onset, indicating that an early treatment could possibly enhance the anti-inflammatory effect of quercetin either in free or liposomal form. The combination of the results obtained for the inflammatory biomarkers highlight that both free and liposomal quercetin were able to attenuate the hypoxia-generated inflammation.

Quercetin has also been described as a compound that exhibits antioxidant properties [14,35]. Since IR is also characterized by the development of oxidative stress [2], the role of quercetin treatment in the generation of reactive oxygen species (ROS) was also investigated in this work. However, the methodology used was not able to detect a high production of ROS in cells subjected to hypoxia for 10 h, impairing the quantitative analysis of ROS by fluorescence microscopy (Supplementary Materials Figure S1). The data from Supplementary Materials Figure S1 highlight that the 10 h incubation with the positive control (tert-butyl hydroperoxide—TBHP) resulted in a high ROS production in hypoxia that was attenuated during the 4 h reperfusion period, indicating that in the absence of this compound, HepG2 cells were able to respond and decrease the oxidative stress. However, in the absence of the positive control, no significant alterations were detected in ROS production. This could be explained by the high ROS levels production presented by HepG2 cells even under normoxia conditions [36,37,38], which could be masking hypoxia-induced differences in ROS production.

### 3.3. In Vivo Studies

A rat model of hepatic ischemia and reperfusion was developed based on previous published work [2,39]. To access the different phases of the in vivo response in the hepatic IRI model, rats were sacrificed 6 h or 24 h after the reperfusion onset and at each end-time point several parameters were analyzed—serum biochemical parameters, hepatic mRNA expression of inflammatory marker, and liver anatomical characterization. The results obtained served to elucidate the mechanism/responses occurring at each time point to further select relevant analysis time points.

**Figure 9:**
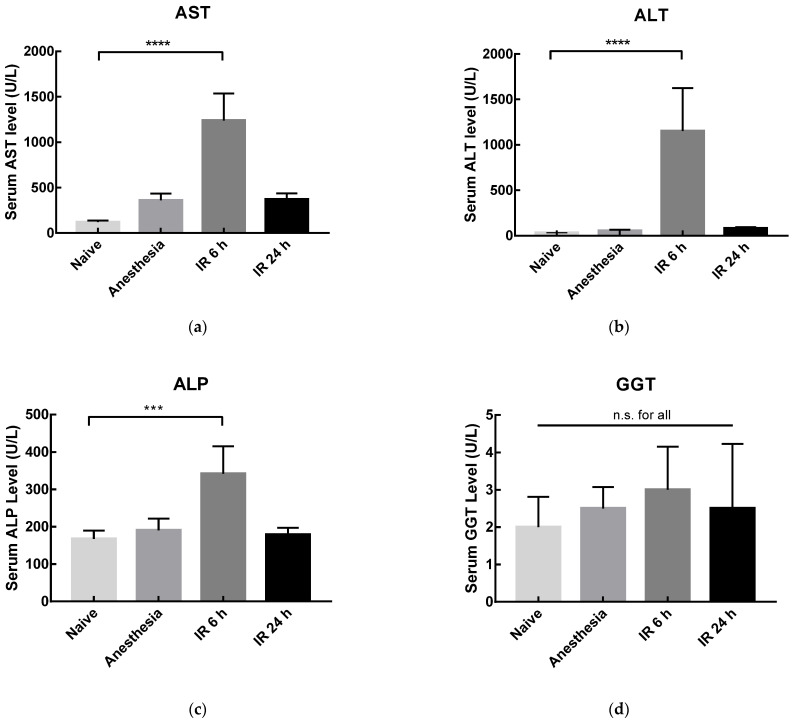
Serum AST (**a**), ALT (**b**), ALP (**c**), and GGT (**d**) levels in rats from naïve, sham anesthesia, or IR groups. Rats were sacrificed 6 h (IR 6 h) or 24 h (IR 24 h) after the reperfusion onset. Results are presented as mean ± SD (*n* = 4). *** *p* < 0.001 and **** *p* < 0.0001 compared with naive group; n.s., statistically non-significant compared with naive group.

Within the biochemical parameters, the level of serum aminotransferases—namely AST, ALT, ALP, and GGT—are considered gold standard clinical biomarkers for liver pathologies [40], and their increased activity has been previously reported in hepatic IRI [41]. Usually, enhanced serum AST and ALT activity levels indicate hepatocytes damage and/or necrosis, while ALP and GGT are more associated with hepatobiliary damage and cholestasis [2]. The hepatic biochemical parameters evaluated in this work at two time points (Figure 9) showed that, when performing the sacrifice 6 h after the reperfusion onset (IR 6 h), a significant increase in serum AST, ALT, and ALP levels was obtained, comparing with the naive group (11-fold, 36-fold, and 2-fold increase, respectively). This result confirmed the successful development of the rat model, since IR was properly inducted. Moreover, it has been reported that AST and ALT present a rapid peak [41,42], which was also observed in the developed model, with an increase in serum levels 6 h after reperfusion onset, and with a decrease in values similar to those of anesthesia group after 24 h. Importantly, this decrease indicates that an acute model of hepatic IRI was achieved, enabling the evaluation of the treatment with quercetin liposomes.

**Figure 10:**
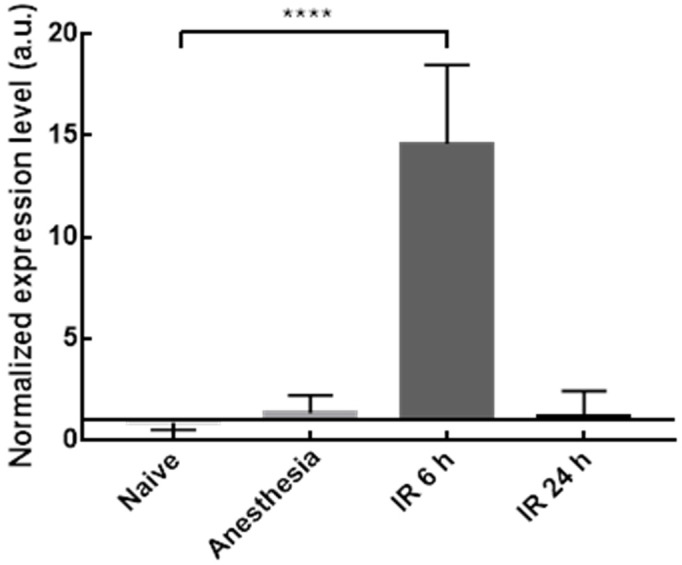
TNF-α mRNA expression level in rats’ liver from naive, sham anesthesia, or IR group. Rats were sacrificed 6 h (IR 6 h) or 24 h (IR 24 h) after the reperfusion onset. Results are presented as mean ± SD (*n* = 4). **** *p* < 0.0001 compared with naive group.

In addition to the hepatic damage, the inflammation progression in the rodent model is also an important parameter to follow. According to the mRNA expression levels depicted in Figure 10, 6 h was the most adequate end-time point for TNF-α mRNA analysis since it was possible to observe a significant over-expression of this biomarker in the IR group compared with the naive group, a feature that characterizes inflammatory conditions [43,44,45,46].

The liver anatomical characterization was performed considering two main groups of indicators: damage and recovery indicators. Focus of necrosis, infarction, and hepatic congestion were included in the first group, and bile duct alterations (e.g., ductular hyperplasia and marginal ductular proliferation) and presence of inflammatory cells were categorized as recovery parameters.

**Figure 11:**
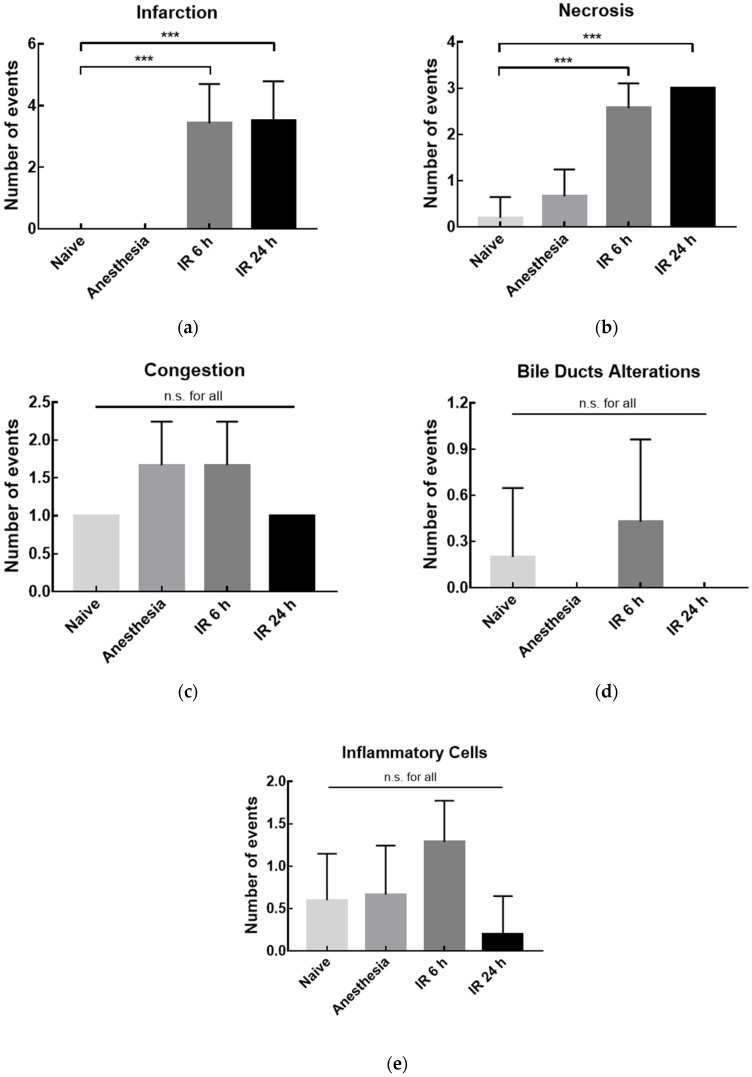
Histochemical analysis of the number of events for rats’ liver from naïve, sham anesthesia, or IR group. Rats were sacrificed 6 h (IR 6 h) or 24 h (IR 24 h) after the reperfusion onset and damage—(**a**) infarction; (**b**) necrosis, and (**c**) congestion—or recovery—(**d**) bile ducts alterations and (**e**) presence of inflammatory cells—indicators were evaluated. Number of events: 0, absence; number, number of detected events. Results are presented as mean ± SD (*n* = 4). *** *p* < 0.001 compared with naive group; n.s., statistically non-significant compared with naive group.

From Figure 11, it is possible to observe altered parameters in rats sacrificed 6 h and 24 h after the surgical procedure. While both end-time points presented significantly higher damage indicators in comparison with the naive group, no alterations were observed in recovery indicators. The differences observed in the damage parameters after 24 h—that were not present in Figure 9; Figure 10—are in line with the expected because the recovery of histological damage was slower than mRNA expression or transaminase level recovery. Since most of the necrosis and infarction events were observed in the liver periphery, the IR induced in animals might not lead to irreversible damage, and hepatic recovery could occur. This hypothesis is in accordance with the results observed for serum parameters (Figure 9) and TNF-α mRNA expression (Figure 10), where significant alterations were observed 6 h after the reperfusion onset but not after 24 h.

Since profiles of inflammation, damage, and recovery were obtained in the animal model with the two end-time points (6 h and 24 h), these experimental conditions were then used in the subsequent experiments to access the in vivo therapeutic effect of the developed quercetin nanoformulation.

**Figure 12:**
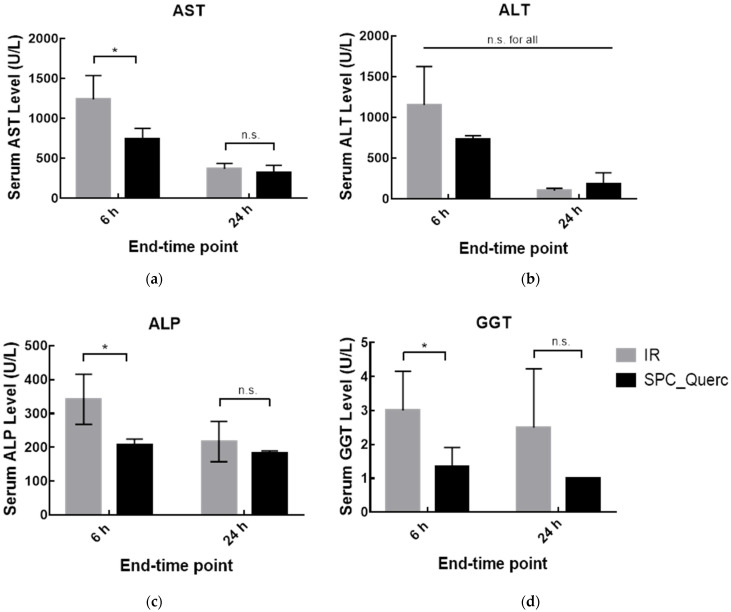
Serum AST (**a**), ALT (**b**), ALP (**c**), and GGT (**d**) levels in rats without treatment or treated with quercetin formulation. Rats were sacrificed 6 h or 24 h after the reperfusion onset. Results are presented as mean ± SD (*n* = 4). * *p* < 0.05 compared with IR group of the correspondent end-time point. n.s., statistically non-significant compared with IR group of the correspondent end-time point.

Data in Figure 12 show that the quercetin nanoformulation was able to reduce serum AST, ALP, and GGT activity levels in rats subjected to hepatic IR, presenting a statistically significant reduction at the 6 h end-time point. At 24 h, since animals had already recovered to naive transaminase levels (non-significant alterations obtained between IR and naive groups—Figure 9), no differences were expected with the treatment. The reduction in serum transaminases levels allows us to conclude that quercetin liposomes were able to attenuate hepatic cell damage caused by hepatic IR.

**Figure 13:**
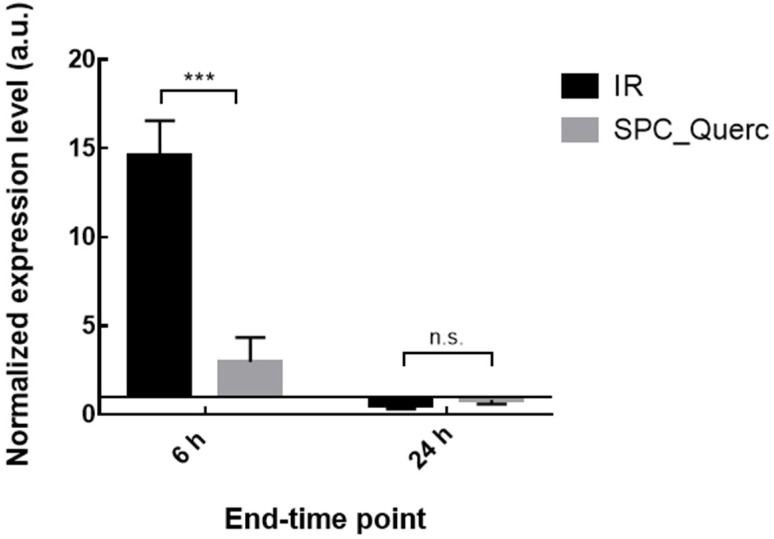
TNF-α mRNA expression level in rats without treatment or treated with quercetin formulation. Rats were sacrificed 6 h or 24 h after the reperfusion onset. Results are presented as mean ± SD (*n* = 4). *** *p* < 0.001 compared with IR group of the correspondent end-time point. n.s—statistically non-significant compared with IR group of the correspondent end-time point.

Additionally, from the RNA expression analysis presented in Figure 13, it was possible to observe a significant reduction in TNF-α level in rats that were treated with SPC_Querc and sacrificed 6 h after the reperfusion onset. This decrease in inflammation suggests an anti-inflammatory role of quercetin liposomes. Twenty-four hours after the surgery, no significant alterations were observed in Figure 10 between IR and naive groups, so a non-significant decrease between quercetin treated and IR group was also anticipated.

**Figure 14:**
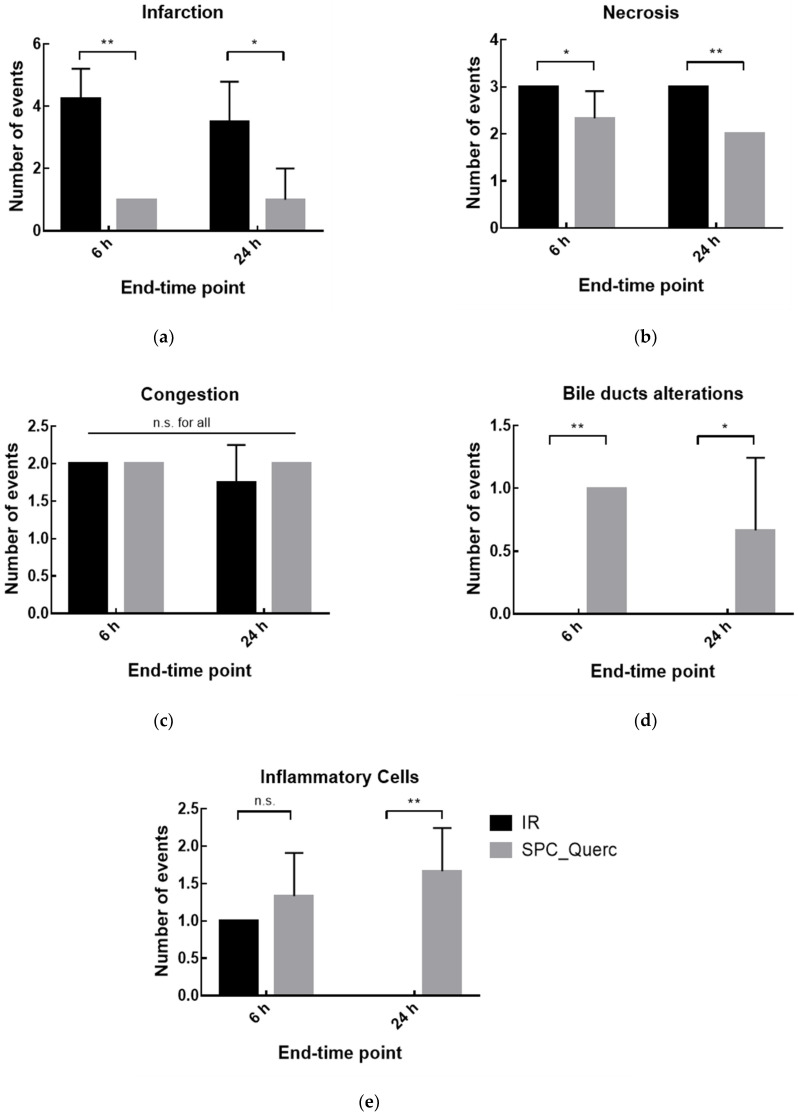
Histochemical analysis of rats liver from IR group or quercetin nanoformulations. Rats were sacrificed 6 h (IR 6 h, SPC_Querc 6 h) or 24 h (IR 24 h, SPC_Querc 24 h) after the reperfusion onset. Damage indicators—(**a**) infarction; (**b**) necrosis, and (**c**) congestion—or recovery indicators—(**d**) bile ducts alterations and (**e**) presence of inflammatory cells—were evaluated. Number of events: 0, absence; number, number of detected events. Results are presented as mean ± SD (*n* = 4). * *p* < 0.05, ** *p* < 0.01 compared with IR group. n.s., statistically non-significant compared with IR group.

Finally, the histological and histochemical analysis for rats treated with nanosystems can be observed in Figure 14, where altered parameters were obtained in rats sacrificed 6 h and 24 h after the surgical procedure. In both end-time points it was possible to observe a reduction in damage indicators upon quercetin liposomes treatment. Moreover, contrary to the data in Figure 11 where no recovery was observed, significantly higher recovery indicators were obtained 24 h after the surgery in the treated groups, demonstrating liposomal quercetin efficacy not only in damage prevention but also in the recovery of hepatic damage after ischemia reperfusion.

Overall, the results obtained with both in vitro and in vivo models unveil a potential anti-inflammatory effect of the developed quercetin nanoformulation that is further improved with liposomes incubation 6 h before the inflammatory onset. The combination of the decrease in the inflammatory process with the lower damage profile and higher recovery observed upon treatment clearly evidence the beneficial effect that a single administration of liposomal quercetin is capable of fulfilling in liver IRI treatment.

When comparing the dose used in the present study (91 mg per administration for an adult with 70 kg) with the regular oral quercetin intake (37.5 to 600 mg/day), the drug delivery system demonstrated advantages in terms of treatment efficacy. Moreover, in comparison with studies from Atef et al. [47], Ali et al. [48], and Uylaş et al. [49] where free quercetin was used to treat hepatic IRI, the efficacious therapeutic effect of quercetin liposomes was herein observed using a 40 to 280 times lower dose than the free drug. This highlights the advantage of the specific hepatic delivery achieved using long-circulating liposomes, which protects quercetin from early metabolization, degradation, and excretion. Another example of a beneficial therapeutic application of quercetin liposomes was demonstrated by Liu et al. [50] for the treatment of other types of acute liver injuries. In that study, a therapeutic effect was observed in transaminases levels and histological parameters, such as in the present work. However, the treatment was administered multiple times (once a day for 1 week and every 2 days for 4 weeks thereafter) and each dose was almost 40 times higher, unveiling a higher therapeutic potential of the quercetin nanoformulation developed in this work.

Several reports have evaluated new approaches for hepatic IRI treatment—either with drugs, nanocarriers, or surgical strategies. However, appropriate comparison is hindered due to two main factors: (i) the selected in vivo model, which differs among laboratories; and (ii) the parameters analyzed to evaluate the therapeutic effect, with distinct combination of techniques being used in each study. Further details about the difficulty in performing comparisons between distinct hepatic IRI treatments can be consulted in Ferreira-Silva et al. [4].

There have not been many reports on the use of nanosystems for the delivery of compounds in hepatic IRI (see [4] and references therein). In fact, a survey of the literature highlights a SOD-containing liposome as the most comparable approach. Therein, the therapeutic effect was similar to that observed for the quercetin liposomes in the present report—namely, a decrease in serum liver biomarkers (e.g., AST and ALT) and an improvement in histological alterations [2,16]. However, since the use of enzymes is associated with higher costs, less complex compounds, such as quercetin, may be advantageous in terms of translation to the clinics.

A quercetin liposomal formulation was previously evaluated for the treatment of other types of acute liver injuries [4]. In that study, a therapeutic effect was observed in transaminases levels and histological parameters. Our study compares positively with those parameters since in those studies the treatment was administered multiple times (once a day for 1 week and every 2 days for 4 weeks thereafter), and each dose was almost 40 times higher than that used in our work. Together, these results show a higher therapeutic benefit from the quercetin formulation herein presented. 

Thus far, the mechanism of action of quercetin in hepatic ischemia and reperfusion injury has not been fully elucidated. Nevertheless, the therapeutic effect of quercetin has been attributed to its antioxidant effect, resulting in a decrease in ROS and RNS production, improved activity of antioxidant enzymes, and restored GSH content [47,51]. Additionally, two other mechanisms—protection of hepatobiliary function and liver structure [47,52] and anti-inflammatory effect—have also been proposed. The latter has been attributed to a decrease in inflammatory biomarkers, such as cytokines, due to quercetin capability to inhibit TNF-α-mediated inflammation (see [11]). Quercetin prevents TNF-α from directly inducing extracellular signal-related kinase (ERK), c-Jun NH2-terminal kinase (JNK), c-Jun, and NF-κB pathways, which are inducers of inflammatory gene expression and protein secretion [11,47,48]. In addition, quercetin may indirectly attenuate inflammation by augmenting the peroxisome proliferator-activated receptor c (PPARγ) action, thereby blocking NF-κB or activator protein-1(AP-1) transcriptional activation of inflammatory genes. Moreover, a crosstalk between NF-κB pathway and ROS levels has been described (e.g., via the alteration in the expression of antioxidant proteins and ROS accumulation), and an antioxidant role of quercetin liposomes might also be occurring [53,54].

In our study, we evaluated these two effects—decreasing of inflammatory biomarkers related to the NF-κB pathway and protective action. In fact, a significant improvement in both parameters after quercetin liposomes treatment could be observed. This shows that the liposomal formulation probably acts via the same mechanism of action than that of free quercetin, with a modulation of inflammatory mediators and molecular pathways of inflammatory-related players, as well as the prevention of liver damage. Our data also show that the nanocarrier did not hinder the therapeutic potential of this flavonoid in hepatic IRI—rather it enabled a dose decrease with similar therapeutic outcome.

## 4. Conclusions

In the present work, stable liposomes were produced presenting high drug concentration and adequate properties for their in vitro and in vivo application, as shown by the several characterization parameters that ensure appropriate stability of the nanosystems.

The hypoxia chamber model was successfully implemented, and a decrease in pro-inflammatory markers expression upon cells incubation with quercetin liposomes was obtained, revealing a potential anti-inflammatory effect of the developed nanoformulation.

The developed in vivo rodent model presents characteristics in accordance with the reported features of hepatic IRI—namely, high levels of serum transaminases activity, elevated levels of mRNA TNF-α expression, and histological alterations [55,56,57].

Six hours after the reperfusion onset was considered, the end-time point presented significant differences between naive and IR groups and provided most of the useful information (biochemical, mRNA expression, and liver damage parameters). Nonetheless, animals sacrificed 24 h after reperfusion onset were also valuable since they provided information about the recovery state of the liver. Altogether, the results obtained indicate a rapid response after hepatic ischemia and reperfusion in which inflammation would be highly present 6 h post-reperfusion onset and would partially recover after 24 h, a profile characteristic of acute pathologies such as hepatic IRI. Finally, data from the biochemical, histochemical, and mRNA expression analysis from rats treated with the nanoformulation revealed an anti-inflammatory role of quercetin liposomes, providing a significant tool for addressing the current bottlenecks in hepatic IRI treatment. 

Additional studies with higher doses or a higher number of administrations of quercetin liposomes would be desirable for understanding whether the therapeutic effect could be further improved. The administration of equivalent doses of free quercetin should also be performed in parallel to evaluate whether the therapeutic outcome was due to the preferential accumulation of long-circulating liposomes in inflamed liver with subsequent release of quercetin in inflammatory sites or whether free quercetin could also exert an anti-inflammatory effect even when low doses are used (in studies from Atef et al. [47] and Ali et al. [48]), 40 to 280 times higher doses of free quercetin were used as a treatment for hepatic IRI, in comparison with the dose used herein). Moreover, it would be interesting to investigate quercetin liposomes’ antioxidant effect in hepatic IRI, which could give further insights into the mechanisms responsible for the positive therapeutic outcome obtained in the present work. This would enable a more complete comparison of the mechanism of action of the quercetin liposomal formulation with the mechanisms of free quercetin described in the literature, for example by Hai et al. [58], Xu et al. [14], and Atef et al. [47]. Taken together, the results obtained demonstrate that the combination of quercetin with a liposomal nanocarrier resulted in an in vitro and in vivo enhanced therapeutic effect, validating the potential of this system for hepatic IRI treatment. Therefore, the employed strategy can be extended to the delivery of other compounds that may have therapeutic potential in hepatic IRI.

## Figures and Tables

**Table 1:** Pair of primers used to amplify human genes by RT-qPCR.

Gene	Forward Primer	Reverse Primer
*HIF1A*	5′–TTG ATG GGA TAT GAG CCA GA–3′	5′–TGT CCT GTG ACT TGT CC– 3′
*IL10*	5′–GGT TGC CAA GCC TTG TCT GA–3′	5′–CCC CCA GGG AGT TCA CAT G–3′
*18S*	5′–GTA ACC CGT TGA ACC CCA TT–3′	5′–CCA TCC AAT CGG TAG CG–3′

**Table 2:** Pairs of primers used to amplify rat genes by RT-qPCR.

Gene	Forward Primer	Reverse Primer
*TNFA*	5′–ACC ATG AGC ACG GAA AGC AT–3′	5′–AAC TGA GAG GGA GCC CA–3′
*18S*	5′–GTA GTG ACG AAA AAT AAC AAT–3′	5′–TTG CCC TCC AAT GGA TCC T–3′

**Table 3:** Full characterization of quercetin nanoformulation (SPC_Querc) and empty liposomes (Unloaded-Lip) according to the lipid composition, the loading capacity (final drug-to-lipid ratio), incorporation efficiency (I.E.), mean size, polydispersity index (PDI), and zeta potential (ζ) at pH 6.0 (*n* = 4).

Nomenclature	Lipid Composition (Molar Ratio)	([Quer]/[Lipid]) f (µg/µmol)	I.E. (%)	Mean Size (µm) (PdI)	ζ at pH 6.0 (mV)
SPC_Querc	SPC:Chol:DSPE-mPEG_2000_ (8:1.5:0.5)	31 ± 3	96 ± 3	0.12 ± 0.01 (0.058 ± 0.010)	−2 ± 1
Unloaded-Lip	SPC:Chol:DSPE-mPEG_2000_ (8:1.5:0.5)	n.a.	n.a.	0.12 ± 0.01 (0.074 ± 0.014)	−2 ± 1

SPC, soybean L-α-phosphatidylcholine; Chol, cholesterol; DSPE-mPEG_2000_, 1,2-distearoyl-sn-glycero-3-phosphoethanolamine-N-[methoxy(polyethylene glycol)-2000]; n.a., not applicable.

## Data Availability

The data presented in this study are available on request from the corresponding author.

## Supplementary Materials

The following are available online at https://www.mdpi.com/article/10.3390/pharmaceutics14010104/s1, Figure S1: Fold change in ROS production in HepG2 cells incubated with 10 µM of free quercetin (Querc) or quercetin liposomes (SPC_Querc) added 6 h before or at the hypoxia onset (simult).
