# Supplementary material for: Quercetin Liposomal Nanoformulation for Ischemia and Reperfusion Injury Treatment
Source: Pharmaceutics. 2022 Jan 3;14(1):104. doi: 10.3390/pharmaceutics14010104 (PMC8779145; doi:10.3390/pharmaceutics14010104)
Supplement: Supplementary file 1 [file pharmaceutics-14-00104-s001.zip › pharmaceutics-1513908-supplementary.pdf]

pharmaceutics

MDPI

Supplementary Materials: Quercetin Liposomal Nanoformulation for Ischemia and Reperfusion Injury Treatment

Margarida Ferreira-Silva, Catarina Faria-Silva, Manuela C. Carvalheiro, Sandra Simões, H. Susana Marinho, Paulo Marcelino, M. Celeste Campos, Josbert M. Metselaar, Eduarda Fernandes, Pedro V. Baptista, Alexandra R. Fernandes and M. Luísa Corvo

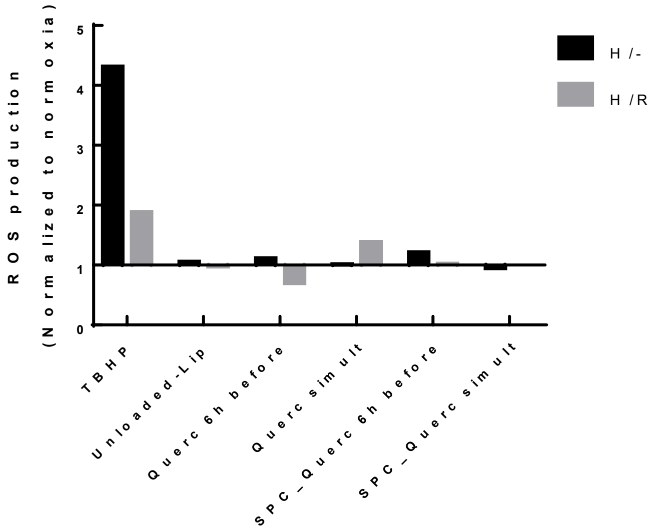

Figure S1. Fold change of ROS production in HepG2 cells incubated with 10 μM of free quercetin (Querc) or quercetin liposomes (SPC_Querc) added 6 h before or at the hypoxia onset (simult). Data was analysed at the end of the hypoxia period (H/-) or after the 4 h reperfusion period (H/R). 100 μM tert-butyl hydroperoxide (TBHP) was used as a positive control for ROS production and unloaded-liposomes (Unloaded-Lip) were used as vehicle control for SPC_Querc. Results from a representative experiment are presented. Each condition was normalized to control cells in normoxia.
